# Characterization of a green *Stentor* with symbiotic algae growing in an extremely oligotrophic environment and storing large amounts of starch granules in its cytoplasm

**DOI:** 10.1038/s41598-021-82416-9

**Published:** 2021-02-03

**Authors:** Ryo Hoshina, Yuuji Tsukii, Terue Harumoto, Toshinobu Suzaki

**Affiliations:** 1 Nagahama Institute of Bio-Science and Technology, Tamura 1266, Nagahama, Shiga 526-0829 Japan; 2 Laboratory of Biological Science, Hosei University, 2-17-1 Fujimi, Chiyoda-ku, Tokyo 102-8160 Japan; 3 Research Group of Biological Sciences, Division of Natural Sciences, Nara Women’s University, Kitauoya-Nishimachi, Nara 630-8506 Japan; 4 Department of Biology, Graduate School of Science, Kobe University, 1-1 Rokkodai-cho, Nada-ku, Kobe, 657-8501 Japan

**Keywords:** Biodiversity, Microbial ecology, Phylogenetics, Taxonomy, Carbohydrates

## Abstract

The genus *Stentor* is a relatively well-known ciliate owing to its lucid trumpet shape. *Stentor pyriformis* represents a green, short, and fat *Stentor*, but it is a little-known species. We investigated 124 ponds and wetlands in Japan and confirmed the presence of *S. pyriformis* at 23 locations. All these ponds were noticeably oligotrophic. With the improvement of oligotrophic culture conditions, we succeeded in long-term cultivation of three strains of *S. pyriformis*. The cytoplasm of *S. piriformis* contains a large number of 1–3 μm refractive granules that turn brown by Lugol’s staining. The granules also show a typical Maltese-cross pattern by polarization microscopy, strongly suggesting that the granules are made of amylopectin-rich starch. By analyzing the algal rDNA, it was found that all *S. pyriformis* symbionts investigated in this study were *Chlorella variabilis.* This species is known as the symbiont of *Paramecium bursaria* and is physiologically specialized for endosymbiosis. Genetic discrepancies between *C. variabilis* of *S. pyriformis* and *P. bursaria* may indicate that algal sharing was an old incident. Having symbiotic algae and storing carbohydrate granules in the cytoplasm is considered a powerful strategy for this ciliate to withstand oligotrophic and cold winter environments in highland bogs.

## Introduction

Mixotrophic protists are reported to live in a wide range of environments^1^, even in highly oligotrophic environments where other photoautotrophic and heterotrophic organisms cannot survive^2,3^. Possible reasons why these protists are adapted to such a harsh environment are (1) there are few large predator animals in such ponds^3^, (2) high UV resistance due to symbiosis shading effect^4^, and (3) mixotrophy allows adaptation to harsh environmental conditions by optimizing the combination of heterotrophic and photoautotrophic organisms in the same organism^1^.

Mixotrophic protists such as *Stentor pyriformis* (algae-retaining ciliate) and *Mayorella viridis* (algae-retaining amoeba) are frequently observed and documented as the dominant protist species in highland wetlands in Tohoku district, Japan, where average winter temperatures remain below freezing for a few months^5^. Even in such harsh conditions, these protists survive in non-freezing locations at the bottom of the pond, but it remains unclear how survival strategies of such protists are related to mixotrophy.

The genus *Stentor* (family Stentoridae, order Heterotrichida) is a relatively well-known ciliate characterized by its lucid trumpet shape. *S. pyriformis* is a poorly described species, although *S. pyriformis* is clearly distinguishable from other *Stentor* species (Table S1). The species was first described in 1893^6^ and then appeared in a microbiota report in 1908^7^. However, its next appearance was not until 1994, in the study on revision of the genus^8^. As described in the original literature, difficulties in the cultivation of this species^6^ may have hindered the research on this species. In Japan, *S. pyriformis* can be found only in highland highly oligotrophic moors, suggesting that intracellular symbiotic algae would help this species of *Stentor* survive in such a harsh environment. In this study, we introduce some unique cell morphology of *S. pyriformis* and the characteristics of symbiotic algae in relation to its life strategy.

## Methods

### Sampling

Water containing dead leaves, twigs, or the remnants of submerged plants was sampled from ponds in Japan. The water sample was brought back to the laboratory at Tokyo and was crudely cultured in Petri dishes. A few days later, *Stentor* cells containing green coccoids within their bodies were often observed. If the green *Stentor* was visible, it was directly collected using Komagome pipette or cup attached to the tip of the rod. We measured hydrogen ion concentration (pH) in some pond samples using URCERI Digital PH Meter (Shenzhenshi Huanhui Dianzishangwu, Shenzhen, China) and electric conductivity (EC) using AquaPro Water Quality Tester AP-2 (HM Digital, CA, USA).

### Culture

Strains of *S. pyriformis* were cultured in 2% KCM medium (160 µg/L KCl, 260 µg/L CaCl_2_, 500 µg/L MgSO_4_ · 7H_2_O; pH 6.9) in Petri dishes (diameter, 9 cm; height, 2 cm) under fluorescent (64 W; height, 20 cm) (12L:12D) or LED light conditions at 25 °C. After multiple trials, *S. pyriformis* was successfully cultured only in low EC medium such as 2% KCM. Its EC was identified to be 1.5 µS/cm. The culture medium was changed once a week; half the volume of culture medium (10–15 mL) was discarded and fresh medium was compensated for the shortfall. *S. pyriformis* were fed a non-photosynthetic cryptophyte, *Chilomonas paramecium*, cultured on *Euglena* medium (2 g/L tryptone, 1 g/L proteose peptone, 2 g/L yeast extract, 1 g/L sodium acetate, 0.01 g/L CaCl_2_) in a 50 mL polypropylene tube until stationary phase, which was centrifuged and washed with pure water or by 2% KCM before feeding.

### Cytological observations

For electron microscopy, cells were chemically fixed with glutaraldehyde and osmium tetroxide or by metal contact quick freezing as described previously^9,10^. After thin sectioning, samples on the grid mesh were stained with a lead citrate stain^11^ and threefold diluted EM Stainer (a lanthanoid salts-based stain, Nisshin EM, Tokyo^12^). The presence of α-1, 4-linked glucose in the cytoplasm of the host *S. pyriformis* and in the chloroplast of the symbiont was tested using Lugol’s iodine solution (3% iodine (wt/v), 2% (wt/v) potassium iodine, and 73.4% (v/v) ethanol). Polarized light microscopy using a light microscope (Nikon Eclipse Ni, Nikon, Tokyo) with a set of orthogonal polarizing filters (Nikon) on both the condenser lens and the CCD camera was used for imaging. For Lugol’s iodine staining, 1-μm-thick sections of chemically fixed, Spurr’s resin-embedded samples were stained with Lugol’s iodine solution for 1 min and examined under a light microscope. For comparison, potato starch was stained with Lugol’s iodine solution for 1 min and photographed under the same conditions. For electron microscopy of the iodine reaction, sections were first stained with lead citrate and EM Stainer and then photographed. The sections were further treated with Lugol’s iodine solution for 30 s, and the same field of view of the same sample was photographed again under an electron microscope.

### Reinfection experiment

We investigated whether endosymbiotic algal cells isolated from *S. pyriformis* could also be infected with *Paramecium bursaria* (strain PbKb1) and coexist in the cytoplasm. The reinfection experiment was conducted according to Omura et al.^13^. Aposymbiotic *P. bursaria* was prepared using the method described by Higuchi et al.^14^. When endosymbiotic *Chlorella variabilis* cells isolated from *P. bursaria* is mixed with aposymbiotic *P. bursaria*, they re-establish symbiosis within a few days. Therefore, symbiotic algal cells were isolated from *S. pyriformis* and fed to aposymbiotic *P. bursaria*. After 30 days, microscopic observation was performed to confirm whether *P. bursaria* accepted the alga as a symbiont. *P. bursaria* was fed with *Chlorogonium capillatum* (NIES-3374) once every 3 to 4 days as food.

### DNA extraction, amplification, and sequencing

*Stentor* cells in the fresh sample from Toriko-Daira (the day after the collection) were isolated under a stereoscopic microscope, and each was transferred into a depression slide filled with pure water. Each ciliate was washed through the tip of a micropipette and transferred into another depression, with this process being repeated twice. Before DNA extraction, we cultured these ‘clean’ (without algae outside) ciliates for 2 days. The aim of this short-term culture was to prompt the ciliates to digest the algae, which they had taken as food, not as symbionts. Thereafter, the isolated individuals were washed twice, and then their DNA was extracted. For the cultured *Stentor*, we isolated individuals and washed twice, and then their DNA was extracted. For each strain, about 20 individuals were collected into one sample.

DNA extraction was performed using NucleoSpin Plant II kit (Macherey–Nagel, Düren, Germany) with modified cell fracturing. *Stentor* cells, each containing many algal cells, were incubated for 5 min in 400 µL Buffer PL1 with 10 µL RNase A at 65 °C. After adding 400 µL of glass beads (ø 0.1 mm), each sample was homogenized in BeadSmash 12 (WakenBTech, Kyoto, Japan) at 5,000 rpm for 30 s. The homogenization was repeated five times, and then each sample was again incubated for 10 min at 65 °C. The subsequent procedures were performed according to the manufacturer’s instructions.

PCR was performed to amplify *Stentor* SSU to internal transcribed spacer (ITS) rDNA region using KOD FX Neo (Toyobo, Osaka, Japan) with the primer pair of SR-1^15^ (5′ SSU)/Hits5 (5′ LSU; –GGT TCR CTC GCC GTT ACT A–). The PCR conditions were as follows. An initial denaturation step at 94 °C for 2 min was followed by 45 cycles of the following conditions: 10 s at 98 °C, 30 s at 52 °C, and 90 s at 68 °C. The amplification was completed with a final step of 68 °C for 1 min. The PCR products were verified by agarose gel electrophoresis, cutting out the shorter band (due to shorter ITSs, a general trend in ciliate, and being intron-less) from the gel and purified using NucleoSpin Gel and PCR Clean-up kit (Macherey–Nagel). The above primer pair amplifies ciliate DNA well but not algal DNA as it is very thin. Therefore, algae-targeted PCR was separately performed with the primer pairs of SR-1/INT-5R^16^ (3′ SSU) and INT-4F^16^ (3′ SSU)/HLR3R^17^ (5′ LSU). The PCR conditions were the same as those for *Stentor*. The PCR products were verified by agarose gel electrophoresis and purified using the NucleoSpin Gel and PCR Clean-up kit. The PCR products for both ciliate and algae were directly sequenced.

### Phylogenetic analyses of *S. pyriformis* and their symbiotic algae

SSU rDNA sequences for the *Stentor* species were obtained by searching the keywords [stentor + ssu] and [stentor + 18 s] from the NCBI database. After rough alignment using Clustal X2^18^, the shorter sequences, and sequences including several ‘N’ were removed. Recent phylogenetic analyses including that of *Stentor* species have indicated stable relationships between the *Stentor* species and its sister clades^19–22^. Therefore, the *Stentor* sequences were aligned with a limited number of outgroup taxa. A bootstrap tree was constructed using the neighbor-joining (NJ) method with default setting in Clustal X2 and examined using 1000 bootstrap replicates. For maximum likelihood (ML) and Bayesian inference (BI) analyses, the best nucleotide substitution model for the data set was analyzed using the Akaike information criterion (AIC) via MEGA X^23^, and the GTR + G + I model was selected. ML analyses were performed with MEGA X using the nearest-neighbor interchange (NNI) branch-swapping algorithm and 1,000 bootstrap replicates were used to estimate node support values. BI analyses were conducted using the Markov chain Monte Carlo (MCMC) method implemented in MrBayes v3.2.6^24^. MCMC was run for 10^7^ generations with four chains, and trees were sampled every 1000th generation. The fixed number of samples (25,000) was discarded as burn-in, and convergence was checked by Tracer v1.649.

The SSU rDNA sequences of *S. pyriformis* algae were first checked for group I intron insertions, following the method described by Hoshina^25^. The joined exons were then submitted to BLASTN (NCBI), which indicated that the algae are closely related to species of *Chlorella* clade (Chlorellaceae). The alignment data of chlorellacean SSU + ITS2 rDNA sequences have been published by Heeg and Wolf^26^. Based on this, we added several recently described species, symbionts of some protozoa and sequences obtained here, and then re-aligned them. Tree construction methods (and selected models) were identical to those for the host ciliate, except for MCMC running for 10^8^ generations.

## Results

### Distribution and environment

**Figure 1:**
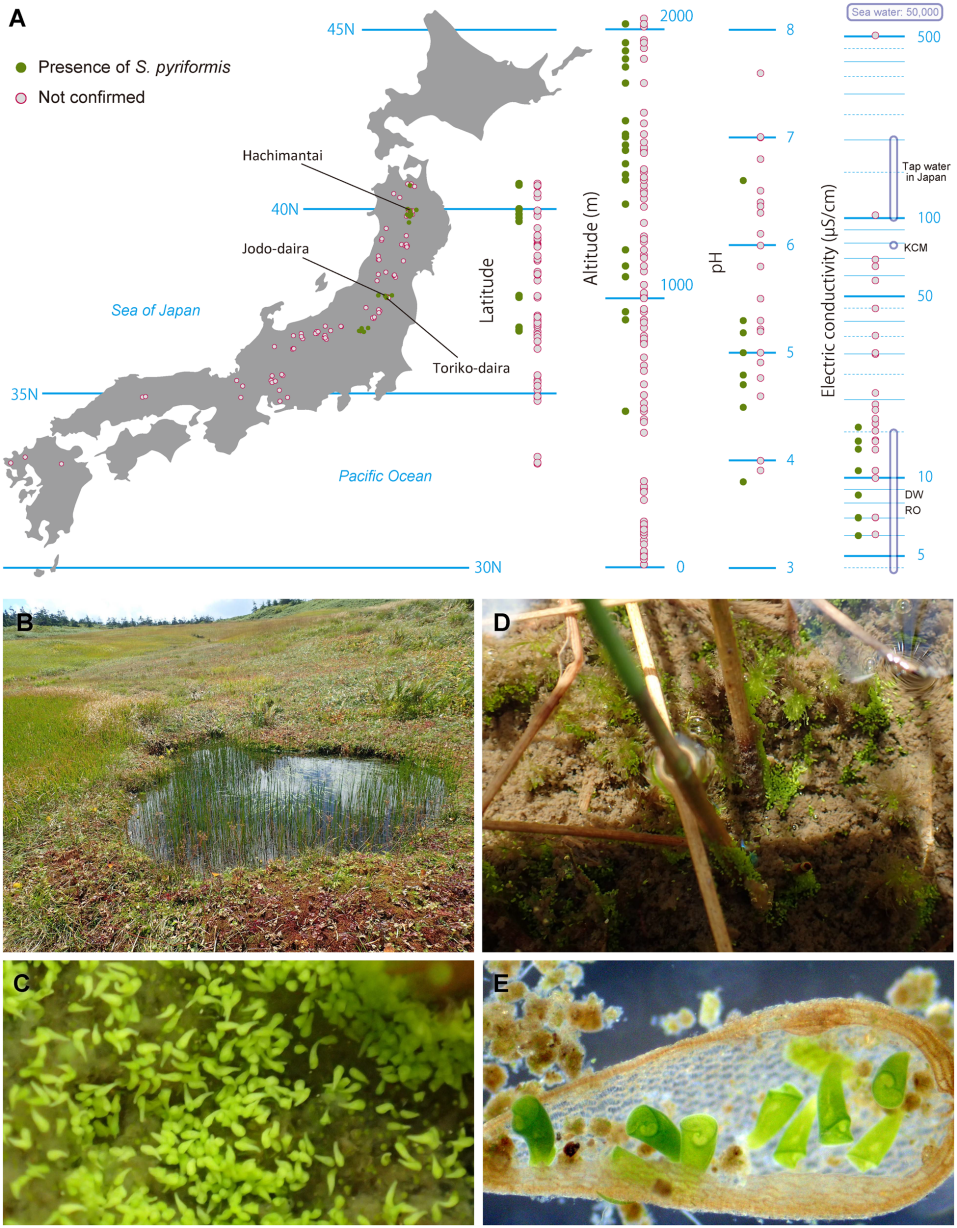
Geographical distribution and habitat of *Stentor pyriformis*. (**A**). Latitude with simple map, altitude, pH, and Electric conductivity (EC) of investigation sites are shown. pH and EC were measured only on 29 sites. For pH and EC, there are some extended notations (e.g., EC: 11–14) in “Tsukii note,” which are spotted as two points of those largest and smallest (e.g., 11 and 14). Reference data of EC values for some general waters were quoted from websites of water companies, Merck Millipore (https://www.merckmillipore.com/), Kurita Water Industries (https://kcr.kurita.co.jp/), and Japan Society of Refrigerating and Air Conditioning Engineers (https://www.jsrae.or.jp/). KCM: 1 × KCM medium (see “Methods”). DW: distilled water. RO: reverse osmosis water. The map data was obtained from Silhouette Design (https://kage-design.com/) and simplified using Adobe Illustrator CS5.B. A bog in Hachimantai area (see Fig. 1). (**C**). Blooming of *S. pyriformis* on the bottom of the bog. (**D**). *Stentor pyriformis* gathering to plant stalks. (**E**). Living *S. pyriformis* gathered in plant debris. A movie is available online showing many *Stentors* on the bottom of the bog at https://1drv.ms/v/s!Aia81H4VPPEYgctoYoHSdbRPYBkvyg?e=KXnnJa/.

We investigated 124 ponds and wetlands in Japan and confirmed the presence of *S. pyriformis* at 23 locations (Fig. 1A). Distribution areas were somewhat biased into four areas of the middle to Northern part of Japan, which are located at 550 to 2020 m altitude. Water conditions were slightly acidic with a pH of 3.8 to 6.6 but showed extremely low values of electric conductivity (EC): 6–16 µS/cm. These EC values overlap with those of distilled water or reverse osmosis water (Fig. 1A). Bogs where *S. pyriformis* was detected were usually located near the mountain peak or along the ridge (Fig. 1B). Frequently, we encountered blooming of *S. pyriformis* on the bottom of the bogs (Fig. 1C). Other times, they were almost all attached to plant stalks or plant debris (Fig. 1D,E).

### Light microscopy

**Figure 2:**
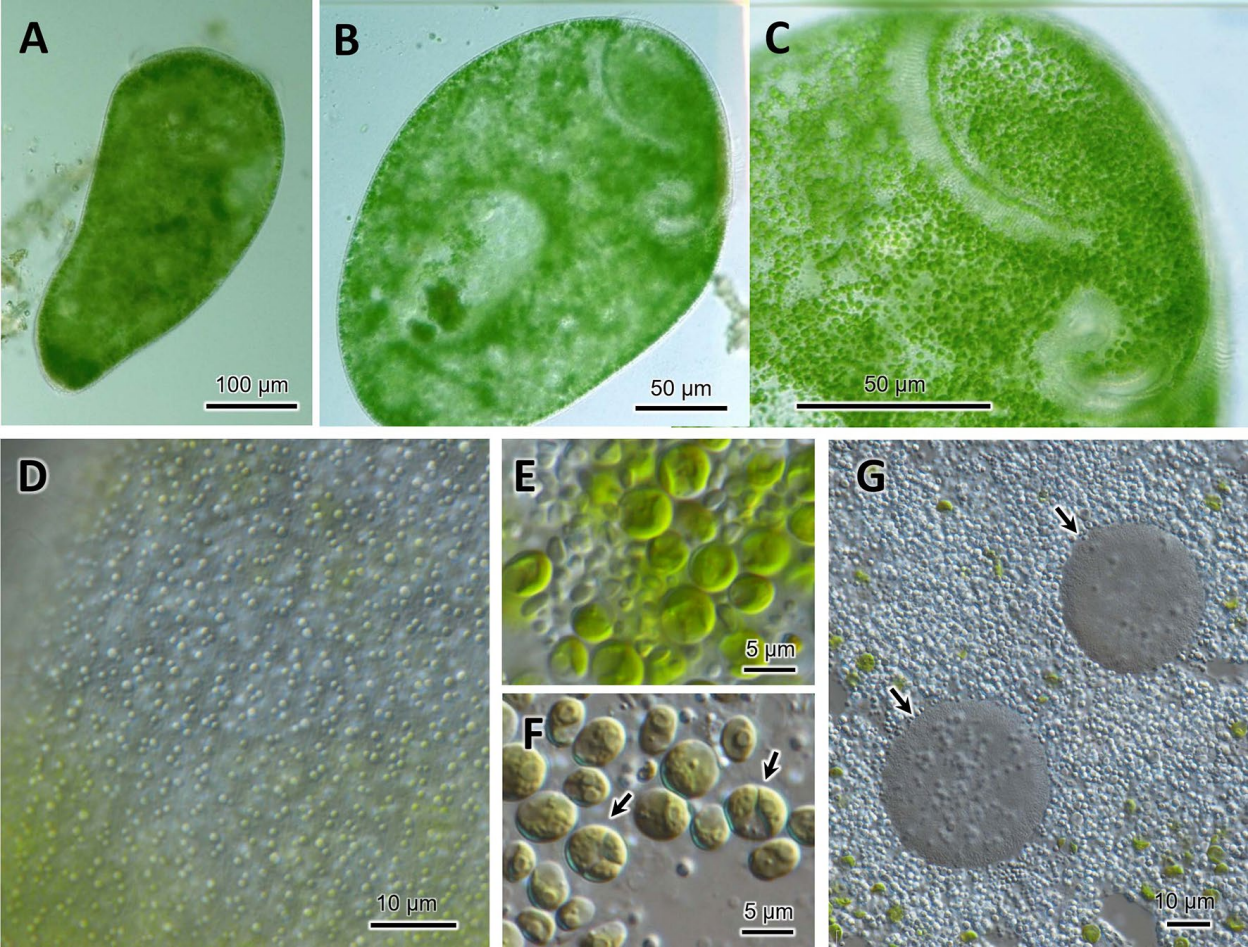
Light micrographs of living and crushed cells of *Stentor pyriformis*. (**A**). A swimming cell showing short and fat body. (**B**). A slightly squeezed cell under coverslip. (**C**). Buccal cavity. (**D**): Surface vesicles immediately under the cell surface. (**E**): Symbiotic *Chlorella* cells as seen in the crushed cytoplasm of *S. pyriformis* with starch granules. The *Chlorella* cells appear vividly green, and dividing cells were rarely seen. (**F**). Symbiotic *Chlorella variabilis* cells in *Paramecium bursaria* for comparison. The picture was taken under the same photographic conditions as (**E**). Cells are pale green and many dividing *Chlorella* cells are observed (arrows). The cell size variation was larger than that in *S. pyriformis*. G. Spherical macronuclei (arrows) found with abundant starch granules in the cytoplasm crushed between the slide and the coverslip.

Cells of *S. pyriformis* were broadly trumpet-shaped, usually 220–500 × 120–300 µm. This length–width ratio did not change significantly between the cells attached to something and swimming (Figs. 1E, 2A). The cells were colored green due to their endosymbiotic green algae that were distributed along the whole body (Fig. 2B,C). A large number of transparent vesicles were present along the ciliary rows immediately under the cell surface (Fig. 2D). To see the contents, the crushed cells were observed. Symbiotic algae appeared to be typical *Chlorella*-like algae, but no dividing alga was observed (Fig. 2E). The algal cells appeared more vividly green when compared to those in *P. bursaria*, suggesting that they are richer in photosynthetic pigments (Fig. 2E,F, and Table S2). The symbiotic algae in *S. pyriformis* had the same size (Table S2, Fig. S1) and morphology as those in *P. bursaria*, but their biological properties were slightly different. As shown in Table S2, *S. pyriformis*’s algae did not grow on agar plates, but could only be cultured in well-aerated liquid media. Reinfection experiments showed that *S. pyriformis*’s algae failed to re-infect the aposymbiotic strain of *P. bursaria*, but those isolated from *P. bursaria* easily re-infected aposymbiotic *P. bursaria*. Macronuclei were, in general, large and spherical (ø 20–35 µm, Fig. 2G). The average number of macronuclei was 6.1 (range 4–10, n = 9) for freshly obtained samples, whereas four-year cultured cells (Table 1) contained only one or two. Micronuclei could not be identified.

**Table 1:** *Stentor pyriformis* strains and GenBank Accession numbers for the host and symbiont rDNA.

Strains	Collection site*	Collection date	Host rDNA	Symbiont rDNA
Hachimantai 1204	Hachimantai	Sep. 24, 2015	LC533384	LC533388
Jodo-daira 1436	Jodo-daira	Oct. 10, 2015	LC533385	LC533389
Toriko-daira 1256	Toriko-daira	Oct. 10, 2015	LC533386	LC533390
(Fresh sample)	Toriko-daira	Oct. 28, 2019	LC533387	LC533391

*See Fig. 1.

### Cellular structure of *S. pyriformis*

**Figure 3:**
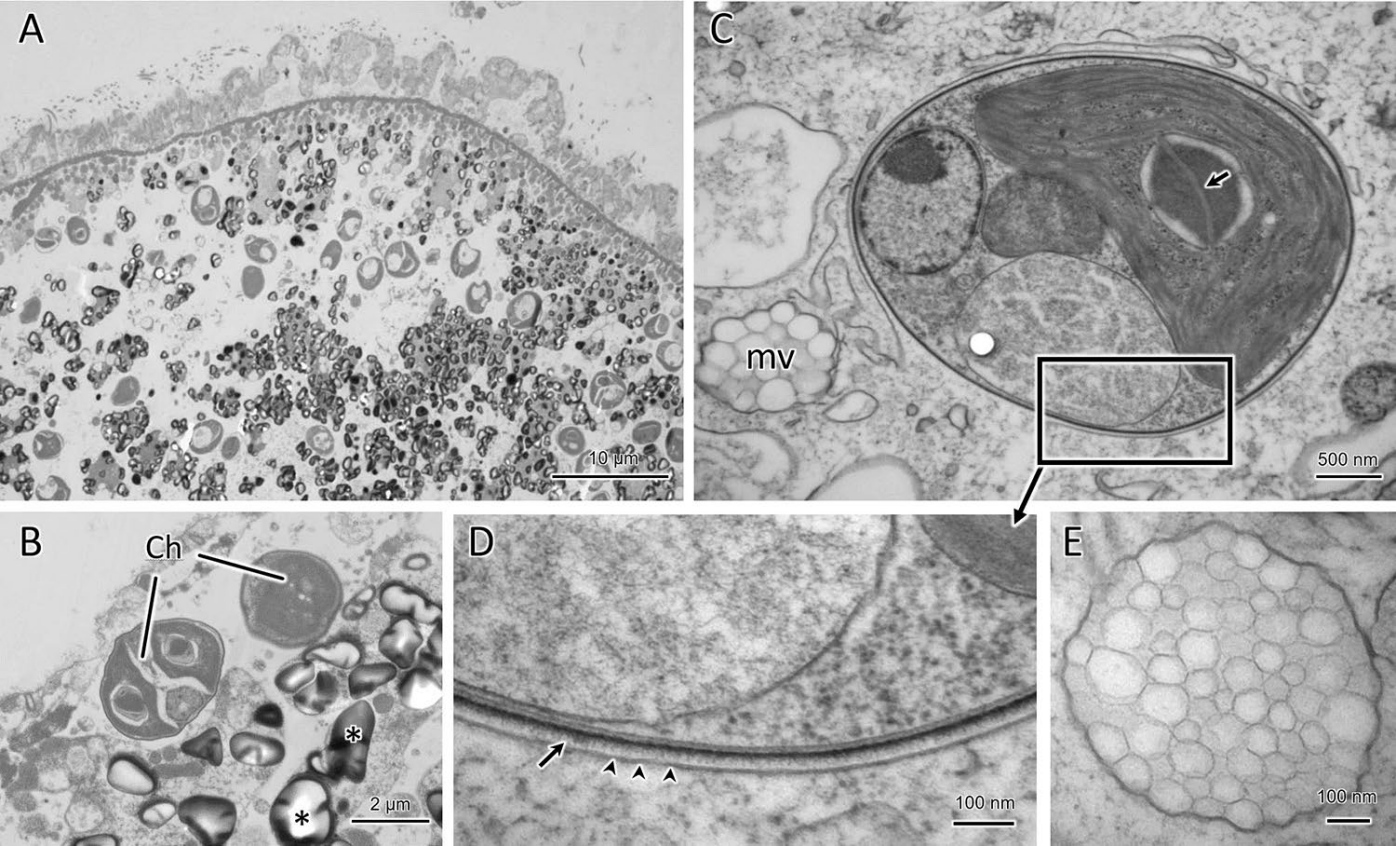
(**A**) and (**B**). Chemically-fixed specimen of *S. pyriformis*. The cytoplasm was observed as highly vacuolated, in which symbiotic *Chlorella* cells (Ch) were observed to be scattered in a large vacuole without being covered by individual surrounding membranes. *Chlorella* at the lower left is a rarely seen dividing organism. Many small electron-dense granules of 1–2 μm in size were present in the cytoplasm. (**C**)–(**E**)S. Quick-frozen and freeze substituted specimen of *S. pyriformis*. The cytoplasm was not vacuolated, and each symbiotic *Chlorella* was surrounded by a symbiosome membrane (arrowheads in **D**). The symbiosome membrane was closely opposed to the cell wall surface by a distance of less than 50 nm. The cell wall surface of the symbiotic *Chlorella* was ornamented by fluffy filaments (arrow). In the symbiotic *Chlorella*, the pyrenoid structure was penetrated by thylakoid membranes (arrow in **C**), characteristic of the genus *Chlorella*. (**D)** is an enlarged view of the area indicated by the rectangle in (**C**). In the cytoplasm, many multi-vesicular bodies of about 1 μm in diameter with unknown function were observed (mv in **C** and also in **E**).

The ultrastructural observations were performed on samples collected on Oct. 28, 2019 at a small pond in Toriko-daira, Japan (37°42′17″N 140°14′53″E). First, the chemically fixed *S. pyriformis* was observed with an electron microscope. Large vacuoles were found inside the cells, and the symbiotic algal cells were inside the vacuoles. The symbionts were found uncovered by individual symbiosome membranes (Fig. 3A,B). Many dark gray stained granules were found in the cytoplasm (asterisks in Fig. 3B). Granules were spherical or oval. The dyeability was not uniform, and the periphery was dyed more intensely. When the same sample was observed by the quick-freezing and freeze substitution method, the appearance in the cytoplasm was observed quite differently (Fig. 3C). Large intracellular vacuoles as observed under chemical fixation were not seen. In addition, individual symbiotic algae were covered by a single symbiosome membrane (Fig. 3C,D). The distance between the symbiosome membrane and the cell wall of alga was extremely close (20–50 nm). Fluffy projections were observed on the cell wall of the symbiotic algae (arrows in Fig. 3D). Pyrenoids were observed in the chloroplasts of the symbiotic algae, through which thylakoid membranes penetrated (arrow in Fig. 3C). Many multi-vesicular bodies were observed in the cytoplasm (mv in Fig. 3C,E). The multi-vesicular bodies were not observed at all in the samples prepared by chemical fixation, suggesting that this structure is very fragile and chemical treatment disintegrates it completely. The number of multi-vesicular bodies per cell was not clear, but there were several granules in each cell. The maximum size of multi-vesicular bodies was about 1 µm, and the size of small vesicles was 100–400 nm in diameter (Fig. S2).

### Cytoplasmic granules

**Figure 4:**
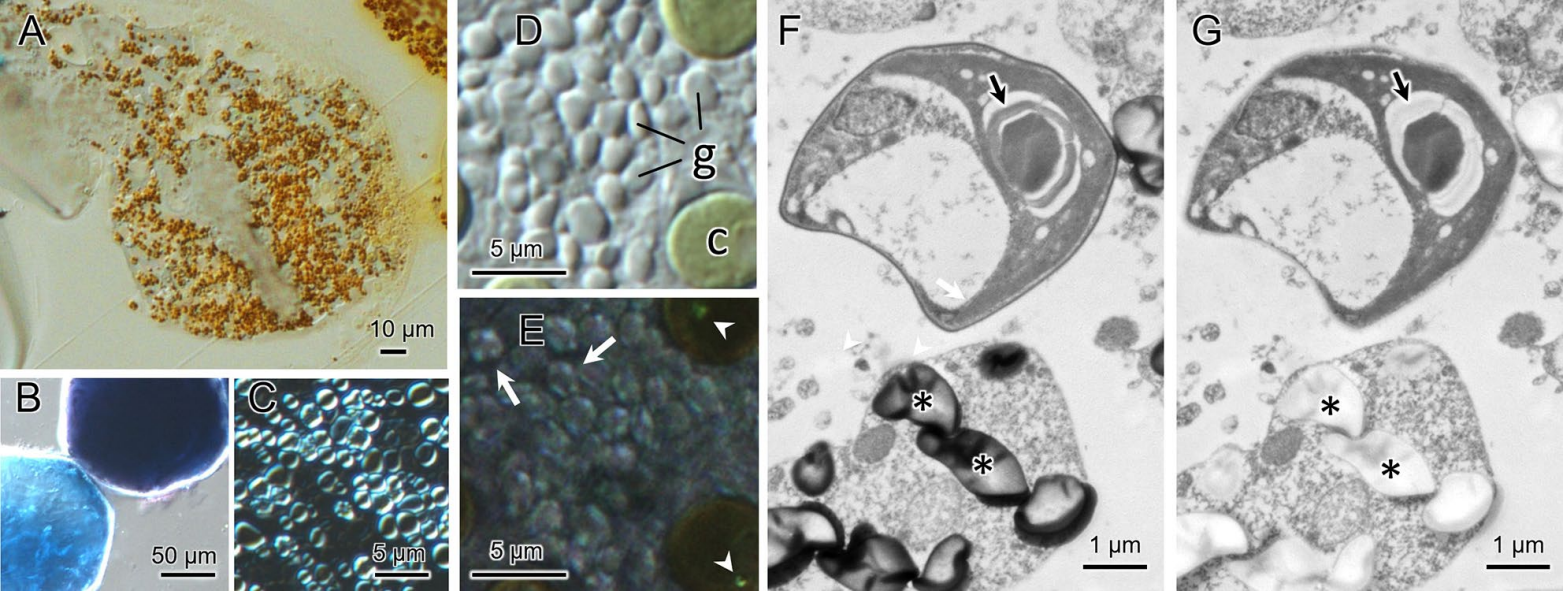
Histochemical localization of starch in *Stentor pyriformis*. (**A**). Light micrograph of a thick section of a Spurr’s resin-embedded cell stained with Lugol’s solution. Cytoplasmic granules stained brown, suggesting high content of α-1, 6-linked glucose branch. (**B**): Potato starch granules stained with Lugol’s solution shown as comparison. Potato starch stains blue due to its high amylose content. (**C**): A DIC image of the freshly isolated cytoplasmic granules observed under the crossed polarizer condition, showing that the granules have a high refractile index. (**D**) and (**E**). Polarization microscopy of starch granules in *S. pyriformis*. (**D**). DIC image of compressed and disrupted cytoplasm of *S. pyriformis*. Symbiotic *Chlorella* (c) and cytoplasmic granules (g) are shown. (**E**). Polarization micrograph of the same area as shown in D under the cross-nicol condition. In the crossed polarizer orientation, birefringent anisotropic specimens such as plant starch grains show a characteristic “Maltese cross” pattern (see Olympus website: https://www.olympus-lifescience.com/en/microscope-resource/primer/techniques/dic/dicphasecomparison/). The cytoplasmic granules of *S. pyriformis* show a Maltese cross pattern (arrows), indicating that the granules have birefringent properties like plant starch. Arrowheads show birefringence signals of the starch sheath in symbiotic *Chlorella* cells. (**F**) and (**G**): Transmission electron micrographs of a section of *S. pyriformis* chemically fixed and stained with lead and lanthanoid. Photographs of the same section were taken before (**F**) and after (**G**) treatment with Lugol’s iodine solution. Arrows indicate the pyrenoid starch sheath, which was stained with heavy metals in F, but the stain was removed by iodine treatment. Granules in the ciliate cytoplasm (asterisks) were also destained by iodine treatment.

Cytoplasmic granules are colored brown by Lugol staining, indicating that the granules contained glucans composed of α-1, 4-linked glucose (Fig. 4A). As summarized in Table 2, the stored carbohydrate granules with α-1,4-linked glucose as a backbone are classified into three types based on their physical and chemical properties, amylose-type starch, amylopectin-type starch, and glycogen. The brown color of the intracellular granules of *S. pyriformis* suggests that these granules are rich in amylopectin. For comparison, potato starch, which is an amylose-rich starch, was stained with Lugol under the same condition and turned blue (Fig. 4B). This indicates that this glucan is of the amylopectin-glycogen type. Observation of the isolated granules with a differential interference contrast (DIC) microscope revealed that the granules had a strong refractive index (Fig. 4C). Figure 4D,E shows DIC (D) and polarization (E) microscopy of the cytoplasmic granules of *S. pyriformis*. In the crossed polarizer orientation, each cytoplasmic granule showed a Maltese cross pattern characteristic of starch granules. An ultra-thin section of chemically fixed *S. pyriformis* showed that the granules were stained with heavy metals including osmium, lead, and lanthanoid ions (Fig. 4F, asterisks). The starch sheath in the pyrenoid of the symbiont was also well stained, as shown in Fig. 4F (arrow). After taking the micrograph, the section shown in Fig. 4F was treated with Lugol’s iodine solution, as shown in Fig. 4G. The stain of both the cytoplasmic granules and the starch sheath was removed by iodine treatment, suggesting that the glucan granules and the starch in symbiotic algae share the same affinity to heavy metals.

**Table 2:** Properties of storage glucans with α-1, 4-linked glucose backbone.

Granule storage substances	Starch (amylose)	Starch (amylopectin)	Glycogen
α-1,6-linked glucose branch	None	At every 20–25 glucose units^61^	At every 10–14 glucose units^61^
Iodine coloration	Blue^62^	Red–purple^62^ or Dull-red^61^	Reddish brown^61^
Particle size	From less than 1 μm to more than 100 μm^63^	10–300 nm (α-particle)^64^	
Maltese cross	Yes^65^	Yes^65^	No^66^

### Host rDNA sequence and phylogeny

SSU, ITS1, 5.8S, ITS2, and 5′ LSU rDNA sequences of four *S. pyriformis* strains were obtained (Table 1). There were 2049 nucleotides, and all four sequences were completely identical, including one C/T mixture (Y) at the tetraloop of helix E23_12^27^ in the SSU rRNA structure (data not shown).

**Figure 5:**
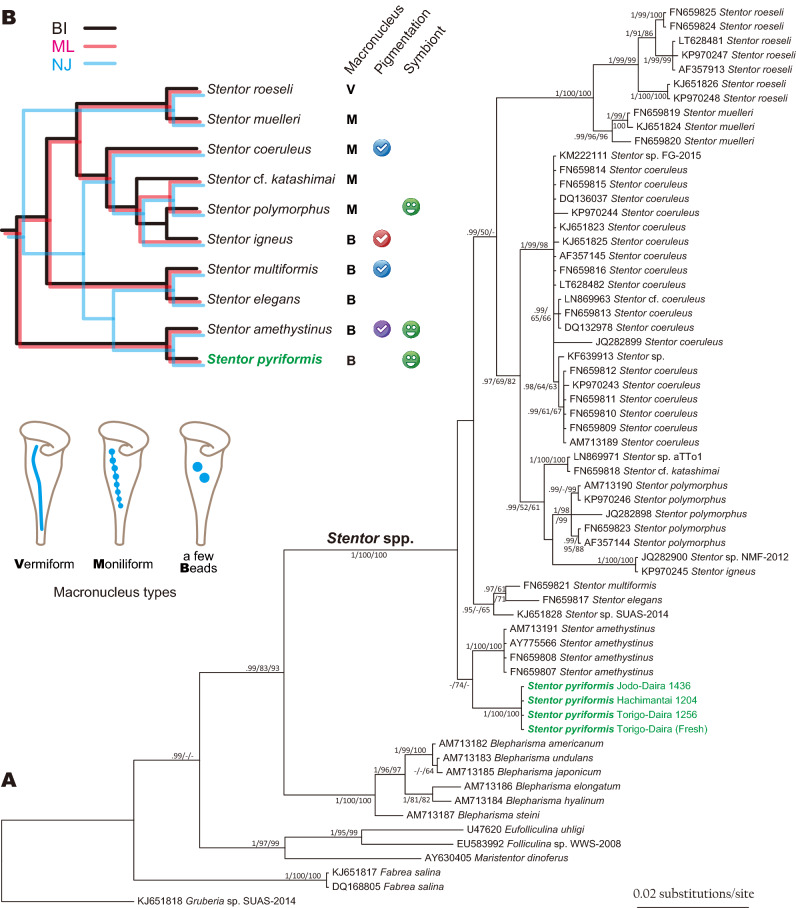
Phylogenetic relationships of *Stentor* species. (**A**). Bayesian inference tree for *Stentor* species based on SSU rDNA sequences. The tree was rooted with *Gruberia* sp. SUAS-2014. Numbers at the branches correspond to MrBayes posterior probabilities (PP)/maximum likelihood/neighbor-joining bootstrap values (BVs). Hyphens correspond to PP values below 0.70 and BVs below 50%. (**B**). Summary of the interspecific relationships and their iconic morphocharacters (shapes of macronucleus, presence or absence of cortical pigmentation and symbiotic algae). The figure explaining the varieties of macronuclei was modified from Foissner and Wölfl^8^.

*Stentor* SSU rDNA were collected and aligned with those of *Blepharisma* and several outgroup taxa. The phylogenetic tree (Fig. 5A) clearly shows the monophyly of the genus *Stentor* (the only genus of family Stentoridae). The monophyly of each species was supported by values of Bayesian posterior probabilities (PP) = 0.99–1 and bootstrap values (BV) = 96–100.

For the branching pattern of the relationships of the *Stentor* species, BI, ML, and NJ analyses showed somewhat different topologies. Here, we provide the species relationships reflecting the differences in these three analyses with iconic morphological characters (Fig. 5B). The monophyletic relationship of *S. roeseli* and *S. muelleri* was perfectly supported. *S. polymorphus*, *S. igneus,* and *S*. cf. *katashimai* (see Thamm et al.^19^) made a clade, which was placed as a sister to *S. coeruleus*. *S. multiformis* and *S. elegans* made a clade. *S. pyriformis* was clustered with *S. amethystinus* in all analyses, although supporting values were not high (PP/MLBV/NJBV = 0.85/74/ < 50). Sequence differences between *S. pyriformis* and *S. amethystinus* were 32 substitutions and 5 indels (Y is counted as one substitution).

### Symbiotic algal rDNA sequence and phylogeny

Algal sequences covering SSU, ITS1, 5.8S, ITS2, and 5′ LSU rDNA for four *S. pyriformis* strains were obtained (Table 1). All sequences contained group I introns at positions S943, S1367, S1512, and L200 (corresponding to the *Escherichia coli* SSU and LSU rRNA). Because of this, each sequence reached more than 3,900 bases (L200 introns were not completely determined). The algal sequences among Jodo-daira 1436, Toriko-daira 1256, and fresh samples from Toriko-daira were identical, even including the introns and the fast evolving ITS rDNA. The algal sequence of Hachimantai 1204 had only one different site from the others. It was at the bulge loop of helix P1 of S1512 intron^28^, where, Hachimantai 1204 had A/T mixture (W), whereas the others had A.

Search for matching sequences using combined SSU rDNA showed they were closely related to the member of *Chlorella* clade (sensu Krienitz et al.^29^), Chlorellaceae (Trebouxiophyceae). Using SSU + ITS2 rDNA of the member of *Chlorella* clade, phylogenetic analyses were performed. All tree analyses (only ML tree is shown) indicated the symbiotic algae of *S. pyriformis* are clustered with *C. variabilis*, with which monophyletic relationships were fully supported (Fig. S3).

## Discussion

### Distribution of *Stentor pyriformis* in Japan and its optimal culture conditions

*S. pyriformis* was described by Johnson in 1893^6^. This algae-bearing *Stentor* has separated spherical macronuclei without pigmentation, which certainly differentiates it from other *Stentor* species (see Table S1, Fig. 5B). While the most common algae-bearing *Stentor*, *S. polymorphus* assumes a slender trumpet shape (often shortened), *S. pyriformis* never resembles such a slender trumpet, but assumes a pear or short conical shape, even when it is swimming^6^. Presence or absence of colored pigmentation is also a prominent characteristic for separating *Stentor* species. Among algae-bearing *Stentor* spp., *S. polymorphus* and *S. pyriformis* only are considered colorless species, whereas colored species are *S. amethystinus*, *S. fuliginosus*, *S. araucanus,* and *S. tartari*^8^ (Table S1). Therefore, *S. pyriformis* is a clearly discernible species; however, it remains underexplored. Indeed, we could only find one paper on the new habitats of *S. pyriformis*^7^, with the exception of the paper of species consolidation of this genus^8^. We confirmed the presence of *S. pyriformis* at 23 locations (Fig. 1A). This indicates that *S. pyriformis* is by no means a rare organism. We assume one of the reasons why *S. pyriformis* has been poorly studied is the difficulty of cultivation. In fact, Johnson^6^ noted that he could not keep them more than a month and never observed any cells in fission. In addition, after five years of failure, it was finally possible to culture *S. pyriformis* for more than several months. Because of objectively unfounded data that we could not include in the distribution data (Fig. 1A), we noticed the wetlands where we found *S. pyriformis* were limited to small ponds or bogs locating near the mountain peak or along the ridge (Fig. 1B). That is, the ponds depending on rainfall without inflowing rivers. Because there is no nutrient flowing in, waters in these ponds showed noticeable oligotrophic tendency, i.e., extremely low electric conductivity (Fig. 1A), which gave us some clues on culture.

The most important point of culture for *S. pyriformis* was keeping the medium lower electric conductivity. We use the KCM medium diluted by 2% with Milli-Q water, and changed medium once a week. A non-photosynthetic cryptophyte, *Chilomonas paramecium* was selected for food. We selected the food so that it would not itself grow in the culture medium. Growing organisms, like photosynthetic algae, seemed to cause damage to *S. pyriformis*. Using this culture method, *S. pyriformis* can be maintained for more than four years (see Table 1). For the organisms not easy to grow in culture, Professor Michael Melkonian mentioned no protist is ‘uncultivable’, there is just human failure^30^. Here, it just became possible to culture *S. pyriformis* 120 years after its discovery; however, this method does not always work. *S. pyriformis* appears to be extremely fragile and disintegrates when any variables are unintentionally altered, that is, the culture is still unstable. When its condition deteriorates, the cells divide unevenly in such a way that a part of the cell is broken. When this happens, the cells become spherical, and the drug drops to the bottom of the dish. It retains this shape for more than a month, but eventually disappears. The doubling time of *S. pyriformis* remains 3 to 4 weeks, even under favorable conditions (data not shown). We occasionally encountered the blooming of *S. pyriformis* all over the bottom of the ponds (Fig. 1C). *S. pyriformis*, therefore, does not seem to be a particularly slow growing species, but our culture method appears to be far from the optimal culture conditions for them. Three *S. pyriformis* strains used in this study are available from the authors upon request.

### Ultrastructure

In this study, we compared the conventional chemical fixation method with the rapid-freezing fixation method for electron microscopic observation. As a result, large vacuoles were observed in the cytoplasm when chemical fixation was used, but not by rapid freezing. Instead, many multi-vesicular bodies were observed in the cytoplasm. The quick-freezing and freeze-substitution method is considered superior in that it can prevent deformation of the intracellular structure compared to chemical fixation^31^. Therefore, it is possible that the originally existing multi-vesicular bodies were artificially disintegrated by chemical fixation, and the constituent biological membranes fused together, eventually forming large vacuoles. To the authors' knowledge, no intracellular structure similar to the multi-vesicular body in *S. pyriformis* has been reported in protists. As multi-vesicular bodies of *S. pyriformis* could only be observed using the freeze-substitution method, similar granules may also be found in other protists if the same technique is used for electron microscopy. In animals, on the other hand, aggregates of secretory vesicles resembling the multi-vesicular bodies of *S. pyriformis* are present in cardiac telocytes^32^. The extracellular vesicles form multi-vesicular structures of about 1 μm in diameter and contain materials for intercellular communication that are involved in cardiac physiology and regeneration. Because *S. pyriformis* cells often form aggregates at the bottom of the pond, some chemicals may be released from the multi-vesicular body, attracting nearby cells and forming aggregates.

Observation by the freeze-substitution method revealed that the symbiosome membrane was in close contact with the symbiotic chlorella. Furthermore, fluffy projections were observed on the cell wall of the symbiotic chlorella. These characteristics were consistent with those of *C. variabilis*, which is symbiotic in the cells of *P. bursaria*^9^. The only difference was that in *S. pyriformis*, the symbiotic chlorella cells were scattered in the cytoplasm, whereas the symbiotic *Chlorella* in *P. bursaria* were anchored directly below the cell surface.

### Storage granules

The iodine in Lugol’s solution selectively binds to α-1, 4-linked glucose found in polysaccharides, such as starch^33^ and glycogen^34^. The color stained with Lugol’s solution reflects the type of glucose polymer. Starches with high amylose content stain blue-violet (cf. Fig. 4B), high amylopectin stains red–purple, and glycogen stains reddish brown (Table 2). The granules in the cytoplasm of *S. pyriformis* stained reddish brown with Lugol’s solution (Fig. 4A), suggesting that these granules are composed of α-1,4-linked glucans with high number of α-1,6-linked branch points, either amylopectin-rich starch or glycogen. The pyrenoid of *Chlorella* spp. is surrounded by a starch sheath of two large plates^35^. As shown in Fig. 4F,G, the image contrast formed by electron staining of the starch granule in the chloroplast (arrow) was lost by treatment with Lugol’s solution. Although the detailed mechanism is unknown, this observation suggests that electron-stained heavy metals (osmium, lead, and lanthanoid ions) bound to the granules may have been eliminated by iodine in Lugol’s solution. The cytoplasmic granules of *S. pyriformis* showed the same staining properties as the starch granules in the chloroplasts of symbiotic chlorella, suggesting that both types of granules share chemical characteristics as polysaccharides.

Alveolates make up one of the most diverse and largest groups of protists. They include three major taxa: dinoflagellates, ciliates, and apicomplexan protozoa. All three alveolate lineages store glucose in an α-1,4-linked glucose chain with α-1, 6 branches. Ciliates are known to synthesize glycogen granules. For example, *Tetrahymena* has glycogen granules between 35 and 40 nm in diameter, each granule being a collection of small γ-granules of 2–3 nm in size^36^. Dinoflagellates and apicomplexans typically produce more complex and larger spherical starch particles, usually greater than 1 μm in size^37,38^. Amylopectin-rich starch and glycogen are very similar polysaccharides, but they differ in granule size and birefringence (Table 2). Starch granules are large, birefringent, and have a high refractive index, but glycogen does not exhibit birefringence, and its granules generally have a size of 300 nm or less. When observed with a polarizing microscope, the starch granules show a Maltese cross pattern. This pattern is derived from the radial arrangement of amylose and amylopectin molecules in granules and is one of the criteria for starch identification. Since the cytoplasmic granules of *S. pyriformis* are large in size (1–3 μm) and show a typical Maltese cross pattern as shown in Fig. 4E, these granules are likely to be starch granules rich in amylopectin.

### Phylogeny of *S. pyriformis* and its morphology

Relationships of *Stentor* species were not clearly resolved. BI and ML analyses indicated basal diverging of the *S. pyriformis* + *S. amethystinus* clade from others, but NJ analysis did not indicate so (Fig. 5). Recent phylogenetic analyses inclusive of *Stentor* species also indicated basal diverging of *S. amethystinus* from the others; however, the monophyly of the others is not highly supported^21,22^. Therefore, the one thing that can be said is that *S. pyriformis* is closely related to *S. amethystinus*.

For the identification of *Stentor* species, the shape of macronucleus, presence or absence of cortical pigmentation, and symbiotic algae are very important and iconic characteristics^8,19^. *S. pyriformis* and *S. amethystinus* share beaded macronuclei and the presence of symbiotic chlorella (Table S1, Fig. 5B). Pigmentation is present in *S. amethystinus,* but not in *S. pyriformis*. Pigmentation is a noticeable characteristic, which tinctures the whole body of *Stentor* cells. The pigment is thought to function as a defense against predators^39^. However, the kind of pigment compound depends on the species^40^, and the relationship between pigment possession and phylogeny is poor (Fig. 5). Of note, colorless vesicles exist in *S. pyriformis* (Fig. 2D). The short and fat shape is also a common characteristic for *S. pyriformis* and *S. amethystinus*, in this genus with many elongated trumpet shape species^6,8^.

### Symbiotic algae in *S. pyriformis*

**Figure 6:**
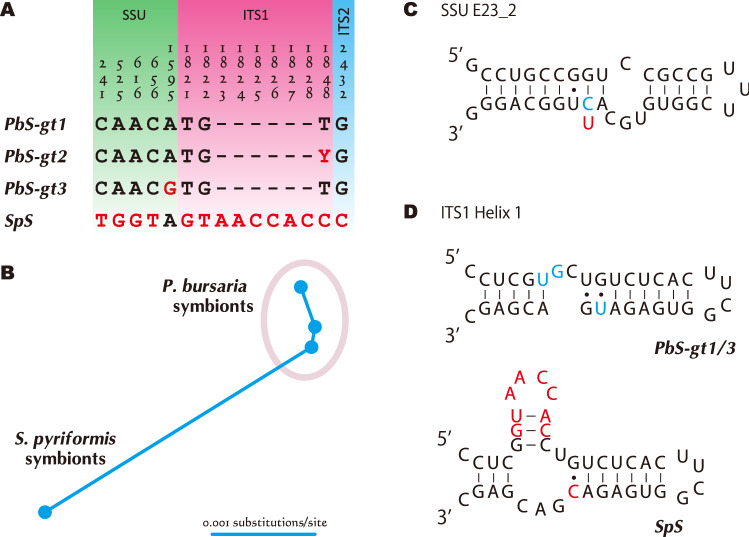
Sequence differences of SSU, ITS1, 5.8S and ITS2 rRNA gene (without group I introns) among *Chlorella variabilis*. “PbS-gt” indicates *Paramecium bursaria* symbiont genotypes. Genotype 1 includes SAG 211-6, ATCC 50258 (NC64A), NIES-2541, and some other US and Japanese strains. Genotype 2 is the alga of Chinese *P. bursaria* strain Cs2, and genotype 3 is the alga of Australian *P. bursaria* strain MRBG1. For further information, see Hoshina et al.^53^. “SpS” indicates the algal sequence of *Stentor pyriformis* strains collected from Japan. (**A**). Different positions. Numerals represent the nucleotide number in aligned sequences (2462 aligned sites). (**B**). Distance tree of above four types of sequences. (**C**). E23_2 helix of SSU rRNA structure that includes hemi-CBC at the alignment position 656. (**D**). Deformation of ITS1 Helix 1 associated with the mutations including several nucleotide insertions.

Algae-targeted PCR products from whole cells of *S. pyriformis* were sequenced directly, and clear peaks were obtained for each. This shows that all or nearly all of the algal symbionts in each *Stentor* cell are unified, regardless of samples under long-term culture or nature. In addition, all symbionts were closely related to *C. variabilis* (Fig. S3), which has been known as a representative symbiont of *P. bursaria* (Oligohymenophorea), the model organism of multi-algae retaining protists (MARP^41^) style symbioses. For the chlorellacean species, the diversity of ITS2 sequence comparisons has often been adopted. For two organisms to compare, ITS2 sequence differences (gaps are counted as a fifth character) usually fall either less than 2% for single species or more than 10% for different species^42,43^. This characteristic simply encourages a species concept. The ITS2 sequences of *S. pyriformis* algae differ only by one nucleotide site out of 248 sites from those of *P. bursaria* algae (Fig. 6A), which strongly suggests the symbiotic chlorella of *S. pyriformis* are also *C. variabilis.* Several *Stentor* species retain coccoid green algae^8^ (Table S1), but only three algal sequences have been published. Two algal sequences from *S. polymorphus* belonged to different clades from Chlorellaceae^44,45^. As for the other algal sequence of *S. amethystinus*, the symbiont may belong to Chlorellaceae^46^. This sequence (EF589816) is short (991 bp) and only covers a part of SSU rDNA; therefore, it was not included in our phylogenetic analyses (Fig. S3). The sequence differs from *C. variabilis* with 10 base changes and 3 indels, indicating that it is not *C. variabilis.*

In the case of *P. bursaria-C. variabilis* symbiosis, *C. variabilis* has been shown to be vastly different from other free-living species. *C. variabilis* demands organic nitrogen compounds^47^ and leaks nearly half of the photosynthate to outside algal cells^48,49^. Furthermore, they are sensitive to the *C. variabilis virus* (CvV; so-called ‘NC64A virus’), which is abundant in natural freshwater^50–52^. Therefore, *C. variabilis* should be considered an already evolved species that is unable to survive without the protection of the host cell^53^.

Four *C. variabilis* rDNA sequences obtained from *S. pyriformis* were identical, with the exception of a nucleotide position in the S1512 intron. Here, the regions without group I introns, i.e., SSU, ITS1, 5.8S, and ITS2 rDNA, are compared among *C. variabilis* sequences of *S. pyriformis* and of *P. bursaria*. Several published sequences cover the above SSU-ITS region, of which varieties are shown as *P. bursaria* symbiont genotype (PbS-gt) 1 to 3 (Fig. 6A). Due to the small number of sequences, it is still unknown whether these genotypes depend on (or are related to) their living regions. Genotype 1 was from USA and Japan, genotype 2 was from China, and genotype 3 was from Australia. All available sequences for *S. pyriformis* symbionts were obtained in this study, and they were all from Japan. As a result, all sequences of *S. pyriformis* symbionts were aggregated into a single genotype SpS, which was distantly related to all *P. bursaria* symbionts, including those from Japan (Fig. 6B). Five variable sites are found in SSU rDNA among *C. variabilis* genotypes, of which four are concentrated to that of the symbionts of *S. pyriformis* (SpS) (Fig. 6A). C/T substitution at alignment position 656 will be a hemi-compensatory base change (hemi-CBC) at the E23_2 helix of SSU rRNA structure (Fig. 6C), whereas the other four sites are at single strand regions (data not shown). Mutations (1821–1828) including comparatively large indels were seen in ITS1 region (Fig. 6A). It was found that all these mutations are assembled in helix 1 (for chlorellacean ITS1 structure, see Bock et al.^54,55^). Thermodynamic analysis via Mfold^56,57^ predicted that PbS sequences form linear helix 1 similar to the other chlorellacean species, but SpS sequences including the additional nucleotides may form a dichotomous branching of helix 1 (Fig. 6D).

The group I introns inserted in SSU rDNA of *S. pyriformis* symbionts are identical to those of *P. bursaria* symbionts^28,58^ in terms of numbers (three introns) and insertion sites (S943, S1367 and S1512). The sequences of S943 and S1512 introns are matched more than 99%. However, with respect to the S1367 intron, a large length gap was found (168 nucleotides) at the tip of P8 (Fig. S4). This section has been indicated as a homing endonuclease gene remnant^28^, and those of *S. pyriformis* symbionts are presumed to be a more degenerated form than those of *P. bursaria* symbionts.

At any rate, the symbiotic algae of *S. pyriformis* were found to be *C. variabilis*. Because *S. pyriformis* never lost the symbiotic algae in four years of culture, and all four algae had nearly identical genetic characteristics, the symbiotic relationships between *S. pyriformis* and *C. variabilis* can be regarded as stable, or permanent. Although *S. pyriformis* and *P. bursaria* share *C. variabilis* as their endosymbionts, considering the genetic differences depending on their host species, the sharing event has not happened recently. Symbiont sharing among various host species has also been known for some ciliates^41,59^ (*Carolibrandtia ciliaticola* in Fig. S3), and a script to spread a particular algal symbiont has been suggested^41^. Given the physiological characters of *C. variabilis* (mentioned above), this algal species might be an ideal algal symbiont, and it will be no surprise if the other protists also retained *C. variabilis* as their algal partners. Research on the symbiotic algae that other *Stentor* spp. have and on host and regional dependencies are awaited.

### Adaptation of *S. pyriformis* to oligotrophic environment in highland marsh

In Japan, *S. pyriformis* lives only in alpine ponds (Fig. 1), where the winter is cold, and the surface of the pond is always covered with ice. The water in these ponds has low electrical conductivity (~ 10 μS/cm), and there are few living organisms except *S. pyriformis*, meaning that only little food is available in wintertime. The reason this ciliate is rich in stored carbohydrate granules may be due to its need for nutrients to survive such harsh winter environments.

Preliminary studies suggest that many protists, especially ciliates, may make starch. Large amounts of cytoplasmic granules that show a Maltese cross were observed in chlorella-bearing ciliates such as *P. bursaria*, while only a small amount of such granules was observed in *Euplotes aediculatus, Paramecium caudatum, Blepharisma japonicum, and Tetrahymena pyriformis*. Protists with symbiotic algae seem to produce particularly large amounts of stored carbohydrate granules in the cytoplasm, but the mechanism of starch synthesis may be widely shared by ciliates.

*P. bursaria* has been shown to be more resistant to starvation conditions than the aposymbiotic strain of the same species^13^. Under food-deprived conditions, *P. bursaria* was interpreted to have survived by digesting symbiotic algae. Resting cyst formation and cannibalism are known as other strategies for protozoans to survive starvation conditions^60^. This study suggests that the use of carbohydrate granules stored in cells may be another possible strategy for ciliates to survive harsh environments such as highland oligotrophic bogs.

## Supplementary Information

Supplementary Information.
