# Supplementary material for: Characterization of a green Stentor with symbiotic algae growing in an extremely oligotrophic environment and storing large amounts of starch granules in its cytoplasm
Source: Sci Rep. 2021 Feb 3;11:2865. doi: 10.1038/s41598-021-82416-9 (PMC7859197; doi:10.1038/s41598-021-82416-9)
Supplement: Supplementary file 1 — Supplementary Information. [file 41598_2021_82416_MOESM1_ESM.pdf]

Supplementary Information

Characterization of a green *Stentor* with symbiotic algae growing in an extremely oligotrophic environment and storing large amounts of starch granules in its cytoplasm

Ryo Hoshina, Yuuji Tsukii, Terue Harumoto, Toshinobu Suzaki

Table S1. Summary of some important morphocharacters of algae-bearing *Stentor* species.

Species	Pigmentation	Macronucleus
S. polymorphus	no	Moniliform
S. pyriformis	no	Beads
S. araucanus	Blue-green	Vermiform
S. tartari	Purplish red	Beads
S. amethystinas	Purple	Beads
S. fuliginosas	Brown or red-orange	Beads

Table S2. Comparison of symbiotic *Chlorella variabilis* isolated from *Stentor pyriformis* and *Paramecium bursaria*

		Host ciliate	
Properties		*S. pyriformis*	*P. bursaria**
Cell diameter (mean ± SD)		5.52 ± 0.46 (n=20)†	5.32 ± 0.65 (n=20) †
Appearance		Vivid green	Pale green
Cell growth	on agar plate	no	Yes
	in liquid medium with aeration	yes	Yes
Re-infection to aposymbiotic *P. bursaria*		no	Yes

**Paramecium bursaria* strain PbKb1 [1].

†mean cell diameters are not significantly different by Welch's t-test (p>0.05)

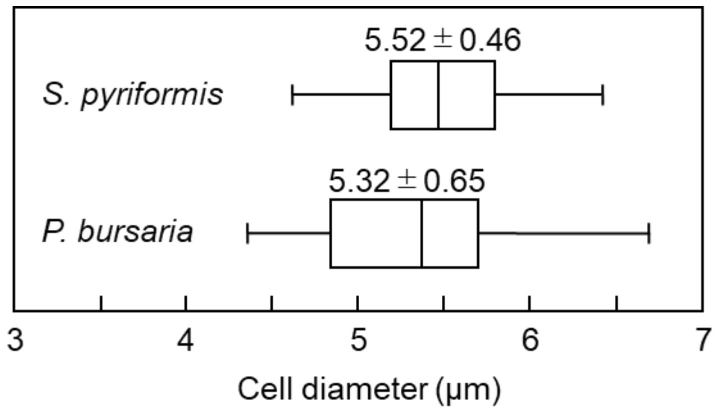

Fig. S1. Boxplot showing the distribution of the cell size of endosymbiotic *Chlorella variabilis* in *Stentor pyriformis* and *Paramecium bursaria*. Boxes indicate the median and interquartile ranges. Vertical lines on the left and right sides of the boxes indicate the minimum and maximum values. The numbers above boxes show mean values and standard deviations (n=20). Mean values are not significantly different ($p>0.05$) according to the Welch's t-test.

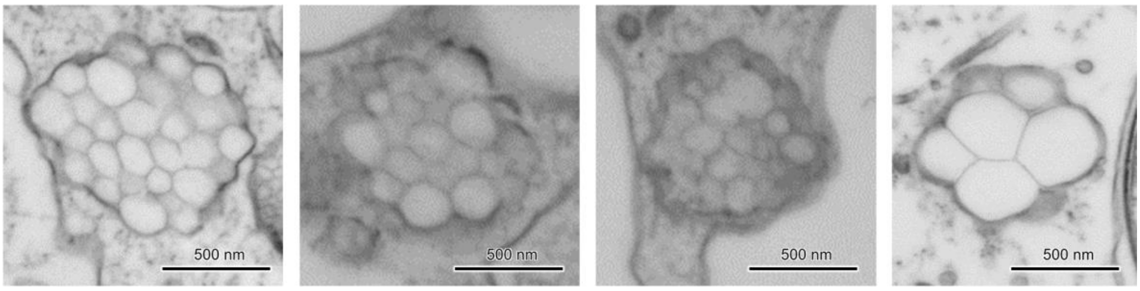

Fig. S2. Multi-vesicular bodies observed in the cytoplasm of *Stentor pyriformis*. The multi-vesicular bodies contained many small vesicles with a diameter of 100-400 nm and were only observed when the cells were prepared by quick-freezing and following freeze substitution with OsO_4. The maximum size of the multi-vesicular bodies was about 1 μm.

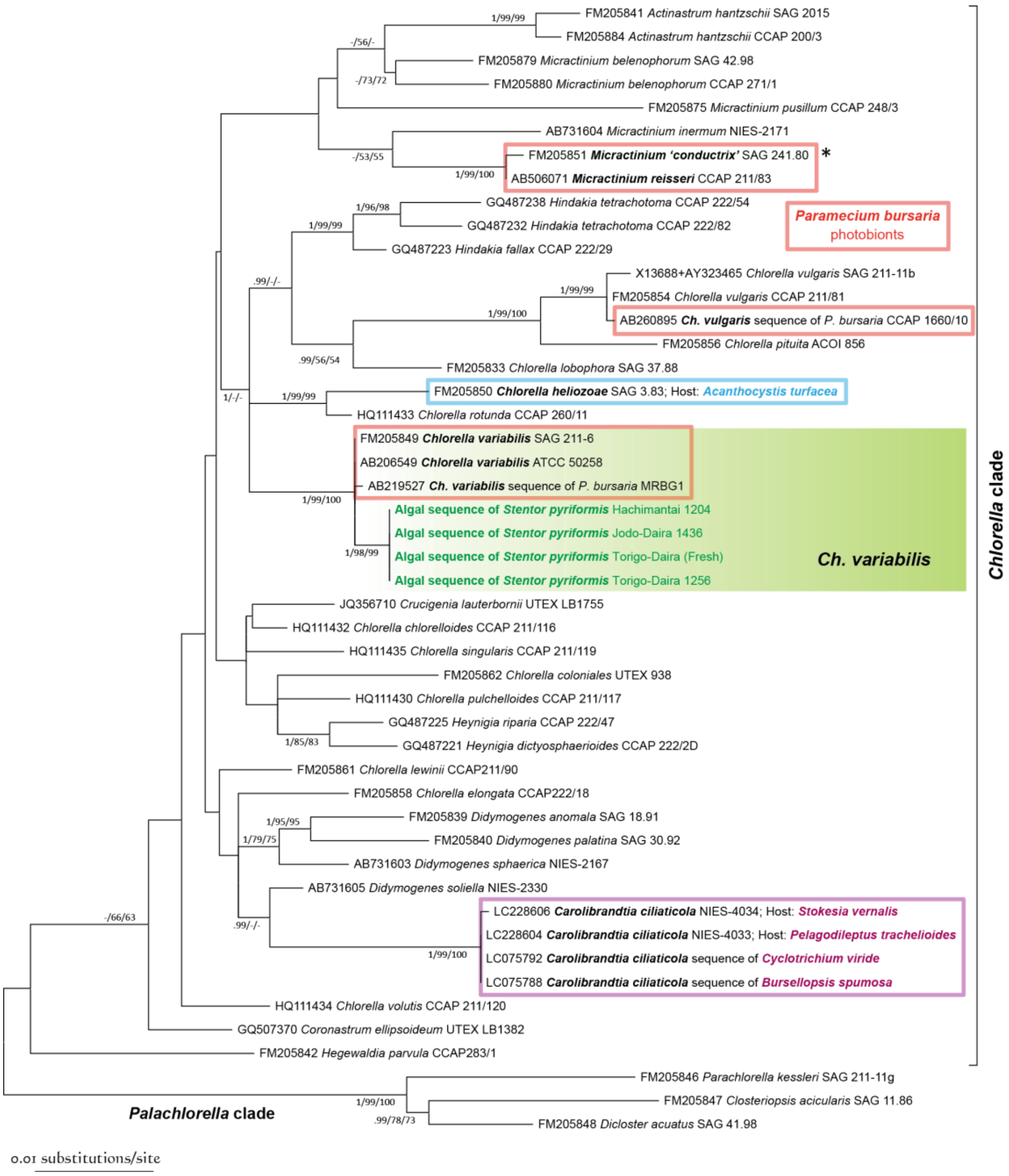

Fig. S3. Phylogenetic position of the symbiotic algae of *Stentor pyriformis*. Maximum likelihood tree based on SSU + ITS2 rDNA is shown. This tree was rooted with three species of *Parachlorella* clade. Numbers at the branches correspond to MrBayes posterior probabilities (PP)/maximum likelihood/neighbor-joining bootstrap values (BVs). Hyphens correspond to PP values below 0.95 and BVs below 50%. Symbiont sequences are boxed. *Chlorella* clade is a treasure trove of symbiotic algae. Recent analyses using SSU + ITS2 rDNA have indicated the polyphyly of the genus *Chlorella* [2–4].

**Micractinium reisseri* and *M. conductrix* are the same species, and there is a dispute over the species name [5].

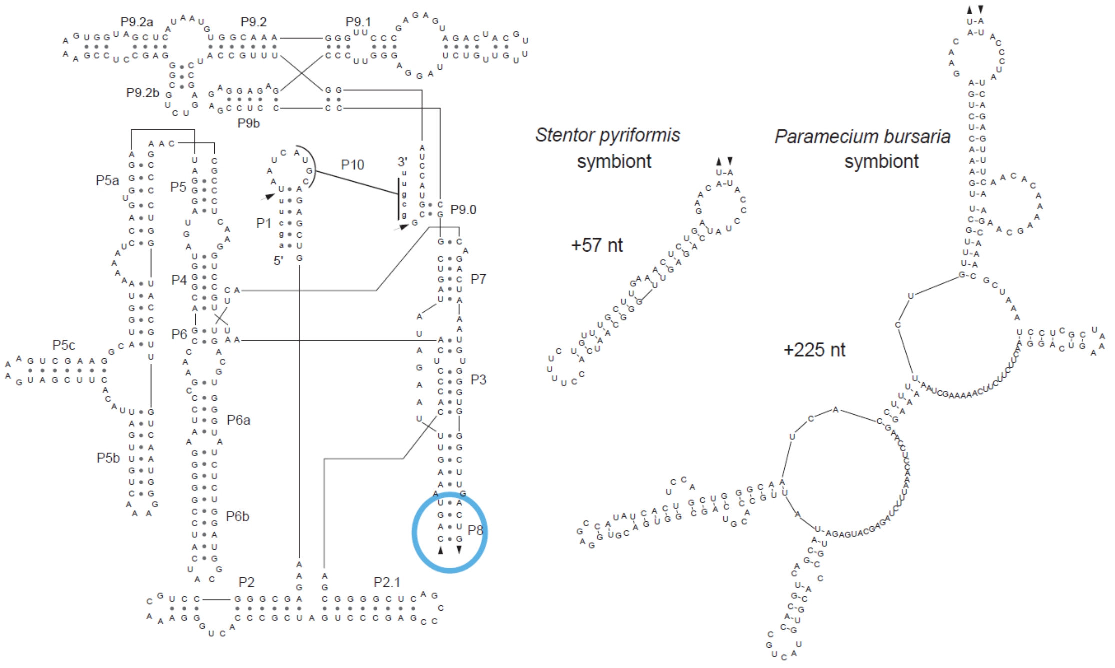

Fig. S4. Secondary structure models for the *Chlorella variabilis* S1367 intron. Base model (left) was modified from Hoshina & Imamura [6]. Capital letters indicate intron sequence and lowercase letters indicate flanking exon sequences. Arrows point to the 5' and 3' splice sites. Pairing segments of P1 to P10 locations are indicated. Sequences beyond the tip of P8 helix (circled) for *Ch. variabilis* of *Stentor pyriformis* and *Paramecium bursaria* were thermodynamically predicted for secondary structures (center and right) via Mfold.

Supplementary References

1. Song C, Murata K, Suzaki T. Intracellular symbiosis of algae with possible involvement of mitochondrial dynamics. *Sci. Rep.* 2017; 7: 1221.
2. Heeg JS, Wolf M. ITS2 and 18S rDNA sequence-structure phylogeny of *Chlorella* and allies (Chlorophyta, Trebouxiophyceae, Chlorellaceae). *Plant Gene* 2015; 4: 20–28.
3. Hoshina R, Kusuoka Y. DNA analysis of algal endosymbionts of ciliates reveals the state of algal integration and the surprising specificity of the symbiosis. *Protist* 2016; 167: 174–184.
4. Pitsch G, Adamec L, Dirren S, Nitsche F, Šimek K, Sirová D, Posch T. The green *Tetrahymena utriculariae* n. sp. (Ciliophora, Oligohymenophorea) with its endosymbiotic algae (*Micractinium* sp.), living in traps of a carnivorous aquatic plant. *J. Eukaryot. Microbiol*. 2017; 64: 322–335.
5. Hoshina R. Comments on the taxonomic treatment of *Micractinium reisseri* (Chlorellaceae, Trebouxiophyceae), a common endosymbiont in *Paramecium*. *Phycol. Res*. 2011; 59: 269–272.
6. Hoshina R, Imamura N. Eu-*Chlorella* large subunit rDNA sequences and group I introns in ribosomal DNA of the paramecian symbiotic alga NC64A. *Phycol. Res*. 2008; 56: 21–32.
